# The effect of anastrozole on bone mineral density during the first 5 years of adjuvant treatment in postmenopausal women with early breast cancer

**DOI:** 10.1186/s40064-015-1096-2

**Published:** 2015-07-01

**Authors:** Hiroaki Inoue, Akira Hirano, Kaoru Ogura, Akinori Hattori, Mari Kamimura, Fumie Okubo, Hiroko Tagawa, Shiho Sakaguchi, Jun Kinoshita, Tadao Shimizu

**Affiliations:** Department of Breast Surgery, Tokyo Women’s Medical University Medical Center East, 2-1-10 Nishiogu, Arakawa-ku, Tokyo, 116-8567 Japan

**Keywords:** Breast cancer, Aromatase inhibitor, Anastrozole, Bone mineral density, Bisphosphonate

## Abstract

**Purpose:**

The administration of aromatase inhibitors is associated with bone loss in postmenopausal women. We assessed changes in bone mineral density (BMD) from baseline to 60 months of treatment in patients receiving anastrozole as initial adjuvant therapy.

**Methods:**

Postmenopausal women with hormone receptor-positive breast cancer receiving anastrozole as adjuvant therapy at our center since 2004 were enrolled in this study. BMD was assessed by dual-energy X-ray absorptiometry at baseline and after 6, 12, 24, 36, 48 and 60 months. Oral bisphosphonate (Bis) treatment was initiated when patients were diagnosed with osteoporosis having a T-score of −2.5 or lower.

**Results:**

Fifty-five patients were enrolled in the study between 2004 and 2011, and the mean follow-up period was 53.6 months. Thirty-five patients were administered Bis (risedronate in 27 patients, alendronate in 8 patients). After 6 months of hormone therapy, BMD decreased by 0.5% from baseline at the lumbar spine (LS) and BMD decreased by 1.5% at the femoral neck (FN). However, BMD increased by 1.9% at the LS and BMD decreased by 1.5% at the FN for 60 months of treatment. In patients treated with upfront Bis (n = 19), 5.4% BMD increase from baseline was noted at the LS whereas in those without Bis (n = 21) BMD decreased by 4.3% from baseline within 24 months (*P* < 0.0001). Fractures were observed in 4 patients (7.3%), and 1 patient (1.8%) had a fragility fracture.

**Conclusions:**

Upfront treatment of Bis with anastrozole significantly increased BMD at the LS and an optimal use of Bis would not increase bone fractures.

**Trial registration:**

UMIN0000017571

## Background

Aromatase inhibitors (AIs) such as anastrozole, exemestane, or letrozole are currently part of the standard endocrine therapy in postmenopausal women with hormone-receptor positive early breast cancer (Goldhirsh et al. 2013; Dowsett et al. 2009). The AIs inhibit the conversion of androgen to estrogen in peripheral fat tissues and tumor cells, which leads to a marked reduction in plasma estrogen (Geisler et al. 2008; Dixon et al. 2008). It is reported that letrozole and anastrozole suppress plasma estrogen by 95.2 and 92.8% (Geisler et al. 2008). However, the suppression of plasma estrogen has been associated with an accelerated rate of bone mineral loss and an increased risk of bone fracture (Simpson and Dowsett 2002; Geisler and Lønning 2008). Not only the Anastrozole, Tamoxifen, Alone or in Combination (ATAC) trial (Eastell et al. 2008), but also the Breast International Group (BIG) 1-98 trial (Zaman et al. 2011) and study of anastrozole with the bisphosphonate risedronate (SABRE) trial (Van Poznak et al. 2010) show that AIs have been associated with bone mineral loss and an increase of bone fracture. Bisphosphonate (Bis) therapy improves bone mineral loss in patients with osteoporosis (Van Poznak et al. 2010; Black et al. 1996; Harris et al. 1999). In almost all reports of bone loss due to AIs, the observation period is 2 years (Van Poznak et al. 2010; Lønning et al. 2005; Goss et al. 2014) and there are few studies of 5 years or longer (Eastell et al. 2008; Zaman et al. 2011). Only one substudy of the ATAC trial indicated the changes of bone mineral density (BMD) for 7 years (Eastell et al. 2008). In view of the assessment for influence of AIs on BMD, it is important to measure BMD without Bis. However, adding Bis is recommended in daily practice for the management of AI-induced bone loss (Hadji et al. 2011). Osteoporotic patients were excluded in the bone substudy of ATAC (Eastell et al. 2008). Thus, we assessed changes in BMD from baseline to 60 months of treatment in patients receiving anastrozole as initial adjuvant therapy adding Bis according to the status of bone loss. This is the first report of the changes of BMD due to 5 years’ treatment of anastrozole for patients including osteoporosis.

## Patients and methods

### Patients

Postmenopausal women with hormone-receptor positive breast cancer receiving anastrozole for 5 years as adjuvant therapy at Tokyo Woman’s Medical University Medical Center East since 2004 were enrolled in this study. All patients had early breast cancer in stage 0–III. Written informed consent was obtained from all patients included in the study.

### Treatment and assessment

All patients received anastrozole 1 mg orally every day. Some patients were prescribed supplementation with Vitamin (D 20 IU/day) and calcium (600 mg/day). BMD for the lumbar spine (L2–L4; LS) and femoral neck (FN) was assessed by dual-energy X-ray absorptiometry (DEXA) at baseline and after 6, 12, 24, 36, 48 and 60 months. Oral Bis (risedronate or alendronate) treatment was initiated when patients were diagnosed as having osteoporosis with a T-score of −2.5 or lower at the LS or FN according to the World Health Organization criteria (World Health Organization 1994). The percentage of changes in BMD from the baseline was calculated for LS and FN. We investigated the changes in BMD for all patients. All patients were divided into the following three groups: patients with upfront use of Bis, those with delayed use of Bis, and those without Bis. The changes in BMD of each group were measured. Recurrent cases were censored at the time of relapse in order to exclude the influence of other therapies (e.g. zoledronic acid or chemotherapy).

### Statistical analysis

The Wilcoxon/Kruskal–Wallis test was used to compare the changes in BMD from the baseline between Bis and non-Bis groups. We analyzed the data of this study using JMP ver 10 (SAS Institute Inc., Cary, NC, USA).

## Results

Fifty-five patients were enrolled in this study between 2004 and 2011. Patients’ median age was 65 years (range 50–85) and the mean follow-up period was 53.6 months (7.2–62.9).

**Figure 1:**
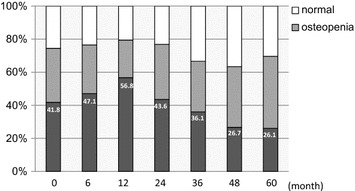
Percentage of osteoporosis. Percentage o f osteoporosis at baseline was 41.8%, the percentage at 1 year of treatment was highest in all patients. After that, osteoporosis decreased gradually.

Seven patients relapsed during the follow-up period. Baseline characteristics and clinicopathological factors are shown in Tables 1 and 2. The percentage of patients with osteoporosis at baseline was 41.8%, and at month 12 it was highest (56.8%) through the entire period. The percentage of patients with osteoporosis gradually decreased in month 24 or later (Figure 1).

**Table 1:** Baseline characteristics

	No. of patients (mean ± SD)	Range
Age (years)	65.2 ± 9.0	50–85
Height (cm)	153.7 ± 5.3	143–165
Body weight (kg)	55.4 ± 8.5	39–78
Body mass index (cm/kg^2^)	23.5 ± 3.4	17.2–32.8

**Table 2:** Clinicopathological factors

	Number of patients (%)
T	
Tis	2 (3.6)
T1	27 (49.1)
T2	23 (41.8)
T3	0 (0)
T4b	3 (5.5)
N	
N0	47 (85.5)
N1–2	8 (14.5)
Estrogen receptor	
Positive	54 (98.2)
Negative	1 (1.8)
Progesterone receptor	
Positive	38 (69.1)
Negative	17 (30.9)
HER2	
Positive	7 (12.7)
Negative	45 (81.8)
Unknown	3 (5.5)

**Figure 2:**
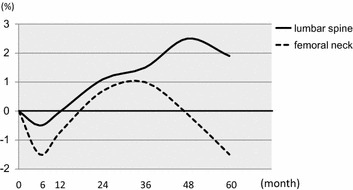
BMD changes in all patients. In all patients, within 6 months of hormone therapy, BMD decreased by 0.5% from baseline at the lumbar spine (*solid line*) and BMD decreased by 1.5% at the femoral neck (*dot line*). However, BMD increased by 1.9% at the lumbar spine and BMD decreased 1.5% at the femoral neck for 60 months of treatment.

Overall, BMD of LS decreased by 0.5% from baseline and BMD of FN decreased by 1.5% at month 6 of hormone therapy. However, BMD of LS increased by 1.9% and BMD of FN decreased 1.5% at month 60 of treatment (Figure 2).

**Figure 3:**
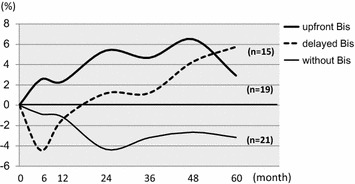
BMD changes of lumbar spine according to upfront, delayed or without Bis. In patients treated with upfront Bis (*bold line*; n = 19), 5.4% BMD increase from baseline was noted at the lumbar spine whereas in those without Bis (*thin line*; n = 21) BMD decreased by 4.3% from baseline within 24 months (p < 0.0001).

**Figure 4:**
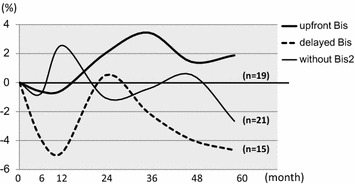
BMD changes of femoral neck according to upfront, delayed or without Bis. In patients treated with upfront Bis (*bold line*), 1.9% BMD decrease from baseline was noted at the femoral neck whereas in those without Bis (*dot line*) BMD decreased by 2.7% from baseline within 60 months (p = 0.1741).

Bis was administered for 34 patients (risedronate for 26 patients, alendronate for 8 patients). Nineteen patients were administered anastrozole with upfront Bis at the same time and 15 patients had Bis added after bone loss (delayed Bis). In patients treated with upfront Bis (n = 19), BMD of LS increased by 5.4% from baseline whereas in those without Bis (n = 21) BMD decreased by 4.3% from baseline at month 24 (*P* < 0.0001). In patients treated with upfront Bis, BMD of LS increased by 2.9% from baseline whereas in those without Bis BMD decreased by 3.2% from baseline at month 60 (*P* = 0.1182; Figure 3). Among the patients treated with upfront Bis, BMD of FN increased by 1.9% from baseline whereas in those without Bis BMD decreased by 2.7% from baseline at month 60 (*P* = 0.1741; Figure 4).

Bone fractures were observed in 4 patients (7.3%), and 1 patient (1.8%) had a fragility fracture (Table 3). Two of them received oral Bis. The annual bone fracture rate calculated from the mean observation period (53.6 months) was 1.6% per year in this study.

**Table 3:** List of fracture cases

Age	baseline T-score	T-score in fracture	Time to fracture (months)	Site of fracture	Cause of fracture
58	Normal	Normal	6	Calcaneus	Trauma
59	Normal	Osteoporosis	26	Tarsal	Trauma
85	Osteoporosis	Osteoporosis	23	Lumber	Fragility
57	Normal	Normal	9	Ankle	Trauma

## Discussion

Anastrozole is superior to tamoxifen in terms of disease-free survival, time to recurrence, and the incidence of contralateral breast cancer in postmenopausal woman with early breast cancer (Baum et al. 2003). However, AIs are definitely associated with the reduction of BMD (Eastell et al. 2008; Zaman et al. 2011). The reduction of plasma estrogen due to AIs has been associated with an accelerated rate of bone mineral loss and an increased risk of bone fracture. However, there is little data on the association between changes of BMD and bone fractures especially for Asian women.

Fractures are related to increased mortality among elderly patients. The mortality after hip fracture is most marked in the first year and thereafter tails off, but always exceeds the mortality of the general population (Johnell and Kanis 2004). Therefore, the prevention of bone loss is important for postmenopausal women in order to preclude a fracture.

Oral Bis therapy increased BMD at clinically important skeletal sites in postmenopausal osteoporosis, and decreased the incidence of bone lumbar and non-lumbar fractures (Harris et al. 1999). The SABRE trial demonstrated that the effect of Bis appeared to be manageable with the use of established practices for maintaining bone health in postmenopausal women treated with AIs (Van Poznak et al. 2010). MA.27B is the largest prospective bone study to assess the role of oral Bis treatment for women with a T-score <−2.0 with concomitant AIs (Goss et al. 2014). These data indicate that Bis prevented AI-induced bone loss in Caucasian women for at least two years.

Our data demonstrated that the Bis (risedronate or alendronate) could control BMD at the LS. However, the effect of Bis at the FN was restricted. In the SABRE trial, the effect of risedronate was demonstrated at the FN (Van Poznak et al. 2010). The effect of Bis at the FN was relatively weak in MA27B. Discordance between these studies is seen regarding the response to Bis at the FN. One reason is that the effect of Bis is essentially larger in LS than FN (Harris et al. 1999). Secondly, the measurement error might be larger at the FN than LS. Furthermore, ethnic difference might influence the response to Bis at the FN. In any case, no femoral fracture was observed in our study. There will be no problem even if BMD of FN does not rise unless the incidence of femoral fractures increases.

Although the observation period of only two studies on AI and Bis in breast cancer (SABLE and MA-27B) is 2 years (Van Poznak et al. 2010; Baum et al. 2003), our study is the result of 5 years’ observation. Our results showed that the BMD of the group receiving Bis at the LS increased during the first 4 years, and afterward decreased. However, the BMD after 5 years increased by 1.9% at the LS from baseline. Our data demonstrated that Bis could control BMD of LS in patients treated with anastrozole for 5 years. Thus, it is not necessary to exclude osteoporotic patients from the treatment of anastrozole.

Which is better, upfront use of Bis for the prevention of osteoporosis or delayed use of Bis after reduction of BMD? Bisphosphonate-related osteonecrosis of the jaw (BRONJ) is a rare, but serious toxicity of Bis. Therefore, we want to avoid the use of Bis as much as possible. This study indicated that BMD of LS increased from baseline at month 60, in patients with both upfront and delayed use of Bis. BMD of FS increased at month 60 in those with upfront Bis; however, it decreased in those with delayed Bis. Thus, it is suggested that upfront use of Bis is recommended for the prevention of decreases in BMD of FN.

Although some data have shown that continuous treatment with Bis for 5 years maintained or increased BMD (Black et al. 2006; Sorensen et al. 2003) in Caucasian postmenopausal women, the BMD decreased from 4 years later in this study. There is a report of the BMD receiving Bis in postmenopausal Asian women. It showed that the pattern of the changes of BMD in Singaporean women was similar to that in Japanese women (Ang et al. 2011).

A review article provided some insights into the potential difference in osteoporosis-related phenotypes between Asians and Caucasians (Lei et al. 2006). These phenotypic differences may partially be the result of different genetic backgrounds, and included the pattern of bone loss and response to treatment.

Bone fractures were observed in 4 patients (7.3%), and 1 patient (1.8%) had a fragility fracture in this study. The annual incidence of vertebral fracture for all Japanese women in their 70 s was 4% per year, and 8.4% per year for those in their 80 s (Fujiwara et al. 2003). The annual bone fracture rate was 1.6% per year in this study, suggesting that Bis would prevent fractures for Japanese women treated with AI. In the ATAC trial, bone fractures were observed in 7.1% of patients over 4 years (Baum et al. 2003). In the BIG 1-98 trial, bone fractures were observed in 9.3% over 5 years (Rabaglio et al. 2009). In view of bone fracture, our results were similar to the results for Caucasians.

This is the first report on changes of BMD in more than 50 patients treated with anastrozole for 5 years including osteoporotic patients receiving Bis.

## Conclusion

In conclusion, our study demonstrated that upfront treatment of anastrozole with Bis significantly increased BMD at the lumbar spine, and an optimal use of Bis would not increase bone fractures.
